# Odin (ANKS1A) Modulates EGF Receptor Recycling and Stability

**DOI:** 10.1371/journal.pone.0064817

**Published:** 2013-06-25

**Authors:** Jiefei Tong, Yaroslav Sydorskyy, Jonathan R. St-Germain, Paul Taylor, Ming S. Tsao, Michael F. Moran

**Affiliations:** 1 Program in Molecular Structure and Function, Hospital for Sick Children, Toronto, Canada; 2 Department of Molecular Genetics, University of Toronto, Toronto, Canada; 3 Department of Medical Biophysics, University of Toronto, Toronto, Canada; 4 Laboratory Medicine and Pathobiology, University of Toronto, Toronto, Canada; 5 Ontario Cancer Institute, University Health Network, Toronto, Canada; Hungarian Academy of Sciences, Hungary

## Abstract

The ANKS1A gene product, also known as Odin, was first identified as a tyrosine-phosphorylated component of the epidermal growth factor receptor network. Here we show that Odin functions as an effector of EGFR recycling. In EGF-stimulated HEK293 cells tyrosine phosphorylation of Odin was induced prior to EGFR internalization and independent of EGFR-to-ERK signaling. Over-expression of Odin increased EGF-induced EGFR trafficking to recycling endosomes and recycling back to the cell surface, and decreased trafficking to lysosomes and degradation. Conversely, Odin knockdown in both HEK293 and the non-small cell lung carcinoma line RVH6849, which expresses roughly 10-fold more EGF receptors than HEK293, caused decreased EGFR recycling and accelerated trafficking to the lysosome and degradation. By governing the endocytic fate of internalized receptors, Odin may provide a layer of regulation that enables cells to contend with receptor cell densities and ligand concentration gradients that are physiologically and pathologically highly variable.

## Introduction

The epidermal growth factor receptor (EGFR) is a prototypical receptor tyrosine kinase (RTK) and functions as part of a network of interacting proteins. Ligand binding is associated with receptor dimerization and activation of the intracellular kinase domain [1]. The EGFR is frequently activated by mutation and/or gene amplification in a variety of human cancers including lung, head and neck, breast, brain, and ovary, and EGFR-expressing tumours frequently evolve to express EGFR ligands (e.g. EGF; transforming growth factor alpha, TGFα) that further promotes their growth [2]. The latent oncogenicity of the EGFR is normally tempered because the activated receptor is subject to down-regulation by endocytosis, culminating with proteolytic destruction in the lysosome [3]. Accordingly, defective endocytic processing of the EGFR is oncogenic (reviewed in [4], [5]). Therefore the EGFR network can drive the cancer cell phenotype subject to a variety of positive and negative regulatory mechanisms acting at the level of the receptor [1], [6]–[8].

RTK signaling and downregulation are highly integrated processes, both largely functions of post-translational modifications (PTMs) and protein-protein interactions. The activated EGFR phosphorylates substrates, including its own C-terminal region, which creates binding sites for a spectrum of proteins with phosphotyrosine (pY)-binding SH2 and PTB domains [9]. EGFR binding proteins include: STAT3, an effector of epithelial-mesenchymal transition [10]; PI3K, an effector of AKT-dependent cell survival [11], which binds through heterodimerized ErbB3 [12] or the adaptor protein GAB1 [13]; and the adaptor proteins GRB2 and SHC, which link to the RAS→ERK axis for cell proliferation [14]. GRB2 is also required for EGFR endocytosis [15], likely through its interactions with the ubiquitin E3 ligase CBL [16], [17]. CBL-mediated ubiquitination of the EGFR is necessary for transport to lysosomes, and GRB2-dependent endocytosis is considered the major pathway of EGFR internalization in many cell types [3], [18]. Temporal analysis of tyrosine phosphorylation by mass spectrometry (MS) applied to EGF-stimulated cells has revealed phosphorylations with rapid kinetics (i.e. reaching maxima within seconds to a few minutes), such as EGFR auto-phosphorylations, which are associated with signal transduction (e.g. ERK activation), and others that accumulate with relatively slower kinetics (i.e. reaching a maxima after 30 min) that are involved in receptor downregulation [10], [19]–[26]. Hence knowledge of the protein-protein interactions and PTMs associated with RTKs and their substrates can provide insight into their functional roles.

Following internalization, the ligand-activated EGFR may recycle back to the plasma membrane, which occurs more or less depending on which of its ligands is bound (reviewed in [27]), or be transported through the endocytic compartment to lysosomes for proteolytic destruction [28]–[31]. EGFR recycling is also triggered in the absence of ligand by cellular stresses such as inflammatory cytokines (e.g. tumour necrosis factor alpha, TNFα) and chemotherapeutic agents including cisplatin in a process dependent on the stress-activated MAP kinase p38 [32]–[35]. Therefore, while many parameters are known to affect the trafficking and stability of EGF receptors, the molecular details of how EGFR trafficking fate is controlled have not been fully defined. These mechanisms are key to controlling the steady state levels of EGFR at the cell surface which are then available to interact with the various EGFR ligands, which themselves may present through a range of concentrations *in vivo*.

**Figure 1:**
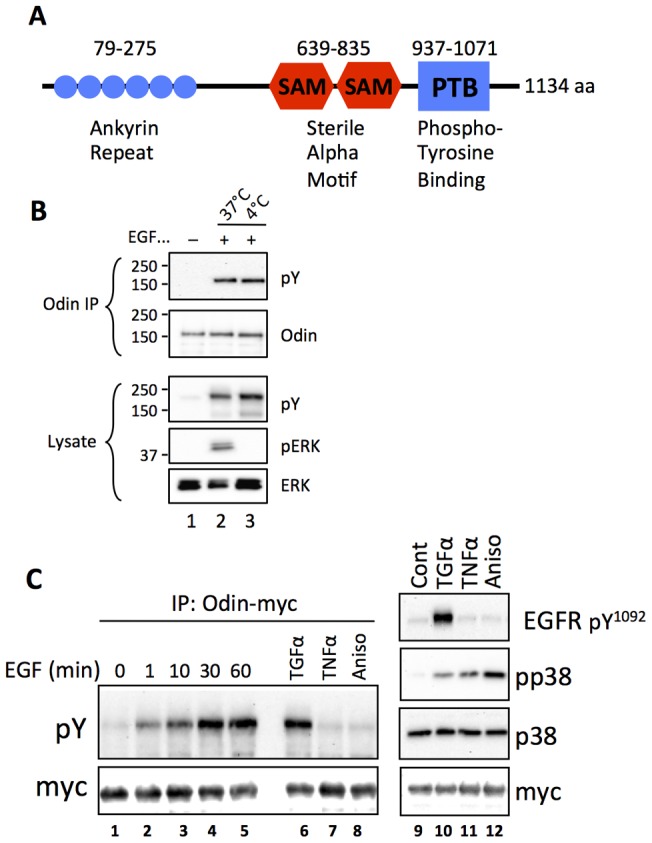
Phosphorylation of the adaptor-like protein Odin in ligand-stimulated and stress-activated cells. A, Schematic drawing of the domain structure of Odin (not to scale) showing amino acid numbers of the indicated features; adapted from Pandey et al. (36). HEK293 cells that stably express EGFR-Flag were subjected to anti-Odin IP of endogenous Odin (B) after treatment with EGF at 37°C or 4°C. The IPs and whole cell lysates were analyzed by Western blot for phosphotyrosine (pY), Odin, and phosphorylated (pERK) or total ERK1/2 proteins (ERK). C, Western blot analysis of anti-myc IPs from the same cells after transient transfection with an Odin-myc expression vector, and treatment with EGF (100 ng/ml) for the indicated durations, and with TGFα (50 ng/ml), TNFα (100 ng/ml), and anisomycin (Aniso; 10 µM) for 15 min. Results shown are representative of three independent experiments.

The ANKS1A gene product Odin was discovered through proteomics analysis as a pY-containing component of the EGFR network [36]. It is widely expressed, and comprised of six amino-terminal ankyrin motifs followed by tandem sterile alpha motif (SAM) domains and a carboxyl phosphotyrosine binding (PTB) domain (Figure 1A). It has not been reported to interact directly with the EGFR *in vivo*, but was implicated as a Src substrate in a colorectal carcinoma cell model [37]. Mice lacking both copies of the Odin gene have no overt phenotype, but *odin^−/−^* mouse embryo fibroblasts (MEFs) have a mildly elevated rate of proliferation in response to EGF and platelet-derived growth factor [38], whereas ectopic over expression of Odin inhibited c-Fos promoter activity [36]. This led to the model that Odin functions as a negative regulator of growth factor RTKs [36], [38]. In a comprehensive, *in vitro* analysis of protein domain interactions with pY sites within the ErbB family of RTKs, the Odin PTB domain was shown to interact with EGFR at position Y998 (the EGFR numbering convention includes the 24-residue signal sequence) [39]. Tyrosine 998 was identified as part of an EGFR internalization motif and implicated in binding clathrin adaptors [40]. Y998 is phosphorylated with relatively slow kinetics following EGFR activation in xenografts and cell lines, and impaired phosphorylation at Y998 or at the proximal phosphorylation site S991 disrupts EGFR endocytosis [19], [41]. Endocytosis component proteins, such as CIN85, were shown by MS to interact with Odin [42]. The guanine nucleotide exchange factor RINL was also shown to interact with the endocytosis factor Rab5 and Odin and thereby affect the degradation of the RTK EphA8 [43]. Odin was itself identified as a ubiquitin-binding endocytosis factor for EphA receptors [44], [45], while its SAM domain was shown to bind directly with the SAM domain of EphA2 [46]. Collectively these data have implicated Odin in RTK endocytosis, but without sufficient mechanistic details to understand its function.

In this study, EGFR trafficking and stability were measured as a function of Odin expression levels. Our findings are consistent with a revised model in which Odin promotes receptor recycling and thereby modulates exposure to ligands and downregulation.

## Materials and Methods

### Constructs and reagents

The murine Odin DNA in the vector pCMV.Sport6 (Origene #BC050847) was sourced from the SIDNET facility (Hospital for Sick Children, Toronto, Canada), and the open reading frame was subcloned into pcDNA3.1/myc-His(−) vector (pOdin-myc). The pGIPZ lentivirus DNA vectors for shRNA nonsilencing control and the shRNA targeting Odin were sourced from SIDNET. The mature sense sequence for Odin shRNA is CAGCAAATAGCAGCATTAA (Open Biosystems Cat#, V2LH_102294). For immunoprecipitation (IP), immobilized mouse anti-myc antibody (9E10), mouse anti-Odin, and mouse anti-EGFR antibodies were obtained from Santa Cruz Biotechnology (Santa Cruz, CA), Abnova (Taipei) and Sigma-Aldrich (St. Louis, MO), respectively. For western blotting, mouse antibodies to Flag and phosphotyrosine (pY; 4G10) were obtained from Sigma-Aldrich and Millipore (Billerica, MA), respectively. Rabbit anti-EGFR antibodies, mouse antibodies to myc (9E10) and GAPDH were obtained from Santa Cruz Biotechnology. For microscopic staining rabbit anti-Rab11 and mouse anti-LAMP1 were obtained from Invitrogen (Carlsbad, CA) and Santa Cruz Biotechnology, respectively. Anti-EGFR-Alexa Fluor^®^ 555 conjugated mouse antibody was from Upstate Biotechnology (Lake Placid, NY). All other antibodies were obtained from Cell Signaling Technology (Danvers, MA). PepClean C-18 Spin columns (Pierce, Rockford, IL) were used for peptide enrichment/washing. All other chemicals were from Sigma-Aldrich, and aqueous solutions were prepared with Milli-Q-grade water (Millipore, Bedford, MA).

### Cell culture, lentivirus-mediated shRNA, and shRNA-resistant constructs

Cells were originally sourced from the American Type Culture Collection (Monassas, VA). Human HEK293 cells were maintained in Dulbecco's modified Eagle's medium (DMEM) supplemented with 10% bovine serum. RVH6849 cells were grown in RPMI 1640 medium supplemented with 10% fetal bovine serum. The HEK-EGFR-GFP line was derived from HEK293, and stably expresses a chimeric EGFR-Flag-green fluorescent protein (GFP) as described previously [47]. HEK293 derivatives stably expressing the fusion proteins EGFR-Flag (HEK-EGFR) or murine Odin-myc (HEK-Odin) were derived by similar methods [47]. All stable cell lines were cultured as described above, and including G418 (400 μg/ml).

Lentivirus expressing Odin shRNA or a non-silencing control shRNA were generated by standard procedures and used to infect HEK293 (shOdin) and RVH6849 cells. Cell cultures were exposed to virus for 8 h, at which time the culture medium was removed and replenished with fresh medium. After a recovery period of 24 h, puromycin (2 µg/ml) was added to select cells with stable virus integration. Cells were analyzed for Odin knockdown after one week of antibiotic selection. A myc-tagged murine Odin DNA construct, resistant to the human-Odin-directed shRNA was transfected to shOdin cells and subjected to G418 selection to generate the “rescue” line srOdin that retains shRNA-mediated knock down of endogenous Odin protein expression, along with ectopic expression of the murine Odin homolog.

### Immunoprecipitation (IP) and Western blotting

For IP and western blotting cells were treated with or without indicated concentrations of EGF for the indicated durations, then rinsed with ice-cold phosphate-buffered saline (PBS) and lysed in NP40 buffer (50 mM TrisHCl pH 7.5, 150 mM sodium chloride, 1% (v/v) NP-40, 100 mM NaF, 1 mM sodium orthovanadate, and protease inhibitors). For immunoprecipitation (IP), clarified whole-cell lysates (1 to 2 mg protein/sample) were extracted with immobilized anti-myc (10 µl) or anti-Odin (1 µg/sample) bound to protein G beads. After 1 h, beads were trice washed with NP40 buffer and proteins eluted by denaturation with Laemmli sample buffer. For western blot, lysates (15 µg protein/sample) or the IP eluates were resolved by SDS-PAGE, transferred to Immobilon-P membranes (Millipore), and then probed with primary and secondary antibodies as described previously [19].

### Cleavable biotin internalization assay

Cells were washed with PBS (pH 8.0) and incubated with Sulfo-NHS-S-S-Biotin (0.5 mg/ml; Pierce, Rockford, IL) in PBS (pH 8.0) for 20 min at 23°C. Excess biotin was quenched with PBS containing 15 mM glycine (pH 7.5). Following treatments, cells were placed on ice, washed, and residual surface-exposed biotin adducts were removed by 3 sequential 8-min incubations with ice-cold glutathione cleavage solution (50 mM glutathione, 75 mM NaCl, 1 mM EDTA, 1% bovine albumin and 0.75% 10 N NaOH). Cells were then washed and lysed. Biotinylated proteins were affinity purified by using streptavidin (SA)-coated beads, and recovered biotinylated EGFR was quantified by anti-EGFR western blotting [35].

### Immunofluorescence staining, confocal microscopy, and flow cytometry

Cells were grown on poly-L-lysine-coated coverslips submerged in a 24-well plate until sub-confluent, and then deprived of serum for 18 h. For synchronized, ligand-pulse experiments, cells were treated with EGF (50 ng/ml) for 30 min at 4°C, washed free of unbound ligand, and exposed to pre-warmed ligand-free medium at 37°C for the indicated time. After treatment, cells were fixed with freshly prepared 3.7% paraformaldehyde in PBS for 20 min at 23°C. Next, cells were permeabilized with 0.2% Triton X-100/5% normal serum in PBS for 10 min. Incubations with the appropriate dilutions of primary (1∶50 to 1∶100 dilution) and Alexa Fluor-conjugated secondary antibodies (as directed by the manufacturer, normally starting as a 1∶200 dilution) were performed in PBS buffer with 0.1% Triton X-100. Confocal microscopy was performed using a Zeiss LSM 510 META laser-scanning microscope (Carl Zeiss, Inc., Thornwood, NY) equipped with a 63× objective. Quantification of colocalization coefficients derived from measured pixel overlap between EGFR and EEA1, Rab11 or LAMP was performed by using Volocity (v6) image analysis software (PerkinElmer) [48]. Mean average values and standard errors were derived from ten independent single cell images from two independent experiments [49].

For flow cytometry, RVH6849 cells at 80% confluence were deprived of serum for 18 h, then suspended by Accutase (Sigma, USA), washed with serum free medium, and then incubate with 50 ng/ml EGF-Alex-Fluo-647 (Invitrogen, USA) at 4°C for 30 min. After wash, the cells were resuspended in 0.5 ml PBS/1%BSA/2 μg/ml Propidium Iodide (PI) solution, and measured by fluorescence-activated cell sorting (FACS).

### Selected Reaction Monitoring Mass Spectrometry

SRM-MS analysis was carried out as described before [19] except, transitions were created and evaluated by using Skyline software [50]. Collision energy (CE) was calculated based on the precursor ion charge state and mass-to-charge ratio using the equations CE  = 0.03*m/z +2.905 and CE  = 0.038*m/z +2.281 for doubly and triply charged precursors, respectively, as described by Maclean et al. [51]. After data acquisition on a triple quadrupole instrument (TSQ Vantage, Thermo Fisher, San Jose, CA), only those peptide transition sets (Table 1, and Fig. S1) adhering to the following requirements were kept: a clear difference between signal and background noise, co-elution of at least 3 transitions and a clear difference between positive (GST-Odin-PTB for Odin) and control (GST for Odin) or synthesized heavy isotope peptide (IPLENLQIIR) for EGFR. The validated transitions for all peptides are listed in Table 1 and were monitored for the duration of the run. Xcalibur, Pinpoint (Thermo Scientific), and Skyline [50] software were employed for SRM data acquisition and total ion current calculations.

**Table 1:** SRM Transitions for protein quantification.

Protein	Peptide	Precursor m/z^1^	Fragment m/z	Retention Time (min)	Ion	Fragment Charge
Odin	NVIAEHEIR	540.793	754.384	14	y6	+1
			683.347	14	y5	+1
			554.305	14	y4	+1
EGFR	IPLENLQIIR (light)	604.872	756.473	14	y6	+1
			998.599	14	y8	+1
			548.330	14	y9	+2
	IPLENLQIIR (heavy)	609.876	766.481	14	y6	+1
			1008.608	14	y8	+1
			553.334	14	y9	+2
GAPDH	GALQNIIPASTGAAK	706.399	1042.589	16	y11	+1
			928.546	16	y10	+1
			815.462	16	y9	+1
			702.378	16	y8	+1
			534.288	16	y6	+1
GST	HNMLGGCPK	507.236	762.364	10	y7	+1
			631.323	10	y6	+1
			518.239	10	y5	+1

1 z  = 2 for all precursor ions.

Cells were lysed in 9 M Urea buffer (20 mM HEPES, pH 8.0, 9 M urea, 1 mM sodium orthovanadate), and then in some instances a defined quantity of a stable isotope-labeled IPLENLQIIR peptide was added to facilitate quantification of the corresponding endogenous non-labeled (EGFR) peptide. The lysates were sonicated for 10 s and then centrifuged for 10 min (20,000×g). The supernatants were reduced with DTT, alkylated with iodoacetamide, diluted 4-fold in 20 mM HEPES buffer (pH8.0), and then digested with trypsin for 18 h at 23°C. The digests were acidified to 1% TFA and then desalted by using a C18 spin column (Pierce, Thermo Fisher) or C18 tip (Eppendorf, Germany) as described previously [19].

### Statistical Analysis

Statistical significance was determined by a two-tailed Student's *t* test. *P* values less than 0.05 were considered to be statistically significant. All western blots and cell images present representative results from experiments that were repeated three or more times, as indicated.

## Results

### The Dynamics of Odin phosphorylation after EGFR activation

In agreement with the findings of Pandey et al. [36], endogenous Odin became tyrosine phosphorylated in response to EGFR activation by cell treatment with EGF (Fig. 1B). This experiment was conducted with human HEK293 that stably express Flag epitope tagged EGFR (45). The extent of Odin tyrosine phosphorylation was similar whether cells were stimulated at 37°C or held at ice-temperature (<4°C) in the presence of EGF, a temperature at which membrane dynamics and EGFR endocytosis are inhibited. The receptor was efficiently activated by ligand at the reduced temperature, as evidenced by its accumulation of pY, but as expected downstream signaling leading to ERK phosphorylation was blocked (Fig. 1B). A kinetic analysis of Odin phosphorylation revealed that Odin tyrosine phosphorylation increased relatively slowly, not reaching an apparent maximum until approximately 30 min after receptor activation, and which was sustained at the 60-min time point (Fig. 1C, lanes 1–5). By contrast, as expected, EGFR auto phosphorylation at Y1092, a GRB2 SH2 domain-binding site linked to Ras→ERK signaling [19], reached a maximum by 1 min post-EGF. A similar high level of Odin tyrosine phosphorylation was observed when cells were treated with TGFα, an EGFR ligand that stimulates receptor recycling [27] (Fig. 1C, lane 6). However, treatment of cells with TNFα or the protein synthesis inhibitor anisomycin, which reportedly induces a cellular stress response that includes p38 MAP kinase-dependent EGFR internalization and recycling in the absence of EGFR activation and auto-phosphorylation [33], [35], effectively activated p38 (see Fig. 1C, lanes 9–12), but did not induce tyrosine phosphorylation of Odin (Fig. 1C, lanes 7–8).

### Quantification of Odin and EGFR by SRM-MS

**Figure 2:**
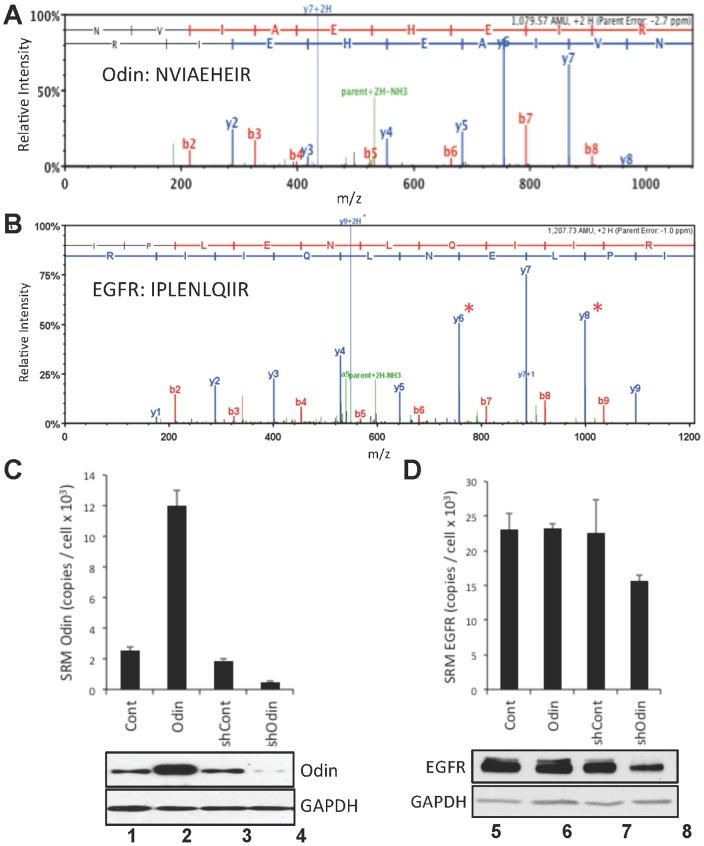
Quantification of Odin and EGFR by SRM. Representative MS/MS spectra of tryptic peptides of (A) Odin sequence NVIAEHEIR (residues 325–333), and (B) EGFR sequence IPLENLQIIR, showing resolved b (red) and y (blue) fragment ions. Odin (C) and EGFR (D) expression level (mean ±SEM, n = 4) in HEK293 cells (Cont), HEK293 stably expressing ectopic Odin (Odin), HEK293 following infection with lentivirus encoding a non-silencing control shRNA (shCont) or lentivirus encoding an Odin-directed shRNA (shOdin) were measured by SRM-MS. The indicated peptides from Odin and EGFR were measured by using the transitions indicated in Table 1 and converted to copies-per-cell by using standard curves developed from measurements of known dilutions of recombinant Odin (see SI Fig. 1), or relative to a spiked-in, stable isotope-containing standard peptide of identical sequence for EGFR. The aligned western blots (15 µg total protein per lane) were probed for Odin or EGFR as indicated, and GAPDH as a loading control. EGFR quantification was performed with enriched membrane preparations (see Materials and Methods).

Odin and EGFR protein levels were measured by SRM MS and verified by western blotting. The precursor-to-fragment SRM transitions are shown in Table 1. Figure 2A shows a representative MS/MS profile of an Odin peptide (NVIAEHEIR, residues 325–333). The co-eluting transitions for the y4, y5, and y6 fragment ions for this peptide were used to quantify Odin, which is summarized in the chart shown in Fig. 2C, along with an aligned western blot that was consistent with the SRM measurements. Quantification of Odin in HEK293 was based on SRM measurements of a defined concentration gradient of a GST-Odin protein (SI Fig. 1). Ectopic Odin expression was approximately 5-fold greater than endogenous, whereas the Odin-directed shRNA, but not the non-silencing control, was associated with an approximate 5-fold decrease in endogenous Odin. The determination of 2500 copies of Odin protein per cell in HEK293 is consistent with recent comprehensive proteome analyses which estimated 7000/cell in Hela cells [52], and 1000/cell in U2OS cells [53]. Figure 2B shows the MS/MS fragmentation pattern of EGFR peptide IPLENLQIIR (residues 99–108), and Fig. 2D shows the SRM-based relative quantification of EGFR in the indicted cell types that have more or less Odin expression. SRM quantification was based on the transitions shown in Table 1, and relative to a spiked-in heavy isotope labeled peptide of identical sequence. From these measurements the level of endogenous EGFR expression was determined as 2.3×10^4^ copies per HEK293 cell (Cont) and similar levels in cells expressing the control non-silencing shRNA (shCont) or over-expressing Odin (Odin). However, EGFR levels were lower by roughly 30% in cells in which Odin expression was knocked down by shRNA expression (shOdin; Fig. 2D, lane 8). The accompanying aligned western blot analysis gave concordant results (Fig. 2D). SRM directed against GAPDH (Table 1) was used to normalize the samples. Note that due to the low expression level of EGFR in the HEK293 cells, more accurate EGFR detection was achieved by conducting the SRM and western analyses with purified crude cell membrane fractions, which recovered >95% of total cellular EGFR and resulting in a >2.5-fold enrichment.

### The involvement of Odin in EGFR trafficking

**Figure 3:**
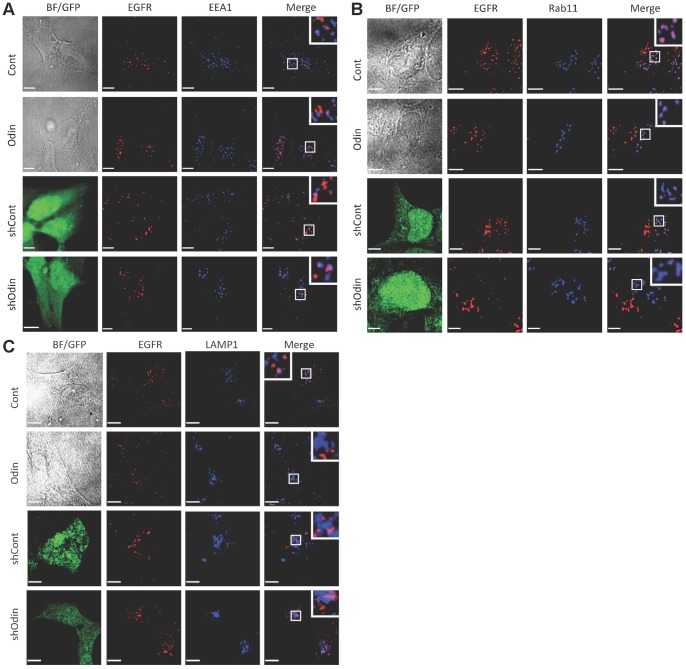
The effect of Odin expression on EGFR trafficking. A–C, EGFR endocytosis after 30 min EGF treatment. Serum-deprived Cont, Odin, ShCont and ShOdin cells were incubated on ice with EGF (50 ng/ml) for 30 min, washed free of unbound ligand, and then provided pre-warmed ligand-free medium at 37°C for 30 min. The first column shows bright field or direct green fluorescence (which identifies lentivirus-infected cells). The cells were fixed and stained with EGFR antibodies (red, second column) and antibodies to EEAI (panel A), Rab11 (panel B), or LAMP1 (panel C) (blue, third column), wherein pink indicates co-localization (Merge, fourth column). Insets show an additional 3-fold magnification of the boxed area. Scale bars are 5 µm.

**Figure 4:**
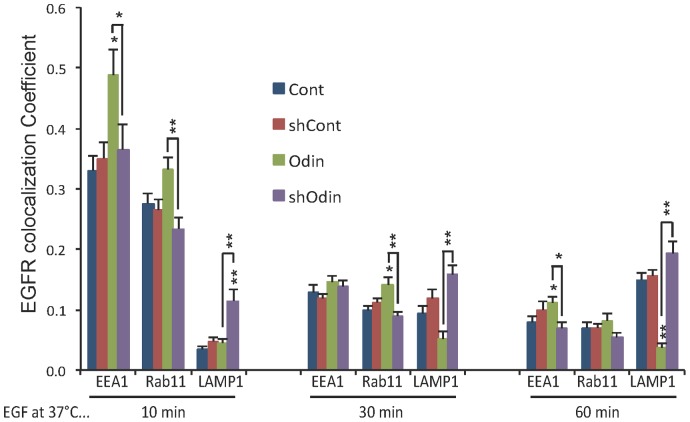
The effect of Odin expression on EGFR trafficking. EGFR co-localization with EEA1, Rab11, and LAMP1, after the indicated incubation time with EGF was quantified by using Volocity software (Perkin Elmer). Co-localization coefficients (mean ±SEM, n = 10) represent pixel overlap between EGFR and EEA1, Rab11, or LAMP1. The coefficient ranges from 0 to 1, with 0 corresponding to non-overlapping images and 1 corresponding to 100% overlap. Asterisks above bars indicate a statistically significant difference from control (Odin v. Cont; shOdin v. shCont); and asterisks above connecting lines indicates the significance of the difference between the connected bars: * *p*<0.05; ** *p*<0.01.

To further address the relationship between Odin and EGFR expression levels and examine EGFR trafficking as a function of Odin concentration, EGFR co-localization with endocytosis markers was examined by cell imaging (Fig. 3). Serum-deprived cells were incubated with EGF at 4°C in order to form ligand-receptor complexes in the absence of endocytosis. Unbound ligand was then washed away, and the cells shifted to 37°C to permit synchronous EGFR internalization and endocytosis [49], [54]–[56]. EGFR and the indicated endocytosis markers: Early endosome-associated protein 1 (EEA1), the recycling endosome-associated small GTPase Rab11, and lysosome-associated membrane protein 1 (LAMP1), were detected by indirect immunofluorescence microscopy (IF) at 10-, 30-, and 60-min time points after a shift in temperature to 37°C. The images in Figure 3 show results for the 30 min time point. The lentivirus used for shRNA transduction encodes GFP, which was visualized by direct green fluorescence (Fig. 3, left columns). EGFR co-localization with the endocytosis markers was quantified by using Volocity software (v6, Perkin Elmer) to measure pixel color coincidence (Fig. 4). At all time points the degree of co-localization of the markers with EGFR was not significantly different between the control HEK293 cells and cells expressing the shRNA control. In all four cell types co-localization of EGFR with the early endosome marker EEA1 and the recycling endosome marker Rab11 was maximal at the 10 min time point, and this was reduced at the 30- and 60-min time points. In the HEK293 (Cont) and shRNA control (shCont) cells, LAMP1 co-localization increased steadily over the three time points. These data are consistent with the expected pattern of EGF-stimulated EGFR endocytic trafficking. In the cells over expressing Odin, the EGFR association with both EEA1 and Rab11 at the 10 min time point, and Rab11 at the 30 min time point, was higher than in the control cells and very statistically significantly higher than in the Odin knockdown cells, and remained higher after 60 min. Conversely, the Odin over-expressing cells showed only minimal co-localization with the late endosome/lysosome marker LAMP1. Even after 60 min the relative lack of LAMP1 co-localization was very significant (p<0.01). By contrast, in the Odin knockdown cells EGFR co-localization with LAMP1 was already very statistically significantly elevated over control levels at the early 10-min time point (*p*<0.01). Furthermore, at the 30 and 60 min time points the difference between the low amount of EGFR-LAMP1 co-localization in the Odin over expressing cells and the elevated amount in the Odin knockdown cells was very statistically significantly different (*p*<0.01).

**Figure 5:**
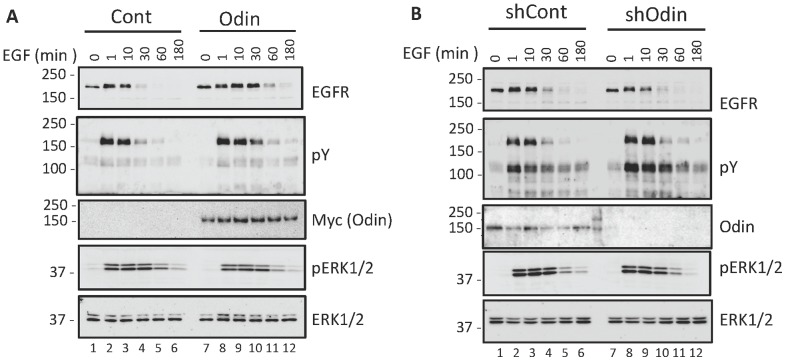
Effect of Odin expression on EGF induced EGFR degradation. A–B, Whole cell lysates from HEK293 cells (Cont), or this cell type stably expressing ectopic Odin (Odin), a control non-silencing shRNA (shCont), or an Odin-directed shRNA (shOdin) were prepared after treatment with EGF (10 ng/ml, 37°C) for the indicated durations, and then western blotted with the indicated antibodies. The results shown are representative of three experiments.

The above results suggested that Odin was affecting EGFR recycling and trafficking to lysosomes. To test this, EGFR levels were measured as a function of EGF treatment and Odin expression. As shown in Fig. 5A, in HEK293 cells expressing low levels of endogenous EGFR, stimulation with EGF resulted in EGFR downregulation as evidenced by the pronounced decrease in EGFR by 30 min post-EGF. By contrast, in HEK293 expressing ectopic Odin the EGFR level was stable at 30 min post-EGF, and decreased at the 60 min time point. EGFR stability was also marginally affected by Odin knockdown by this method of analysis, but in the opposite direction (i.e. faster degradation; Fig. 5B). In the shOdin cells, EGFR degradation was apparent by the 10 min time point, and especially at the 30 min time point were diminished relative to the 1 min signal, and compared to the same time points in the control cells (Fig. 5B). Interestingly, EGF-stimulated activation of ERK, as measured by ERK kinase domain phosphorylation, was not significantly altered as a function of Odin expression levels. At all three levels of Odin expression (basal, ectopic over-expression, knock-down) EGF-stimulated ERK activation was achieved by 1 min, sustained at 10 min, and decreasing by 30 min (Fig. 5). In control experiments the trafficking of the transferrin receptor was unaffected by Odin levels (SI Figures 2–6).

**Figure 6:**
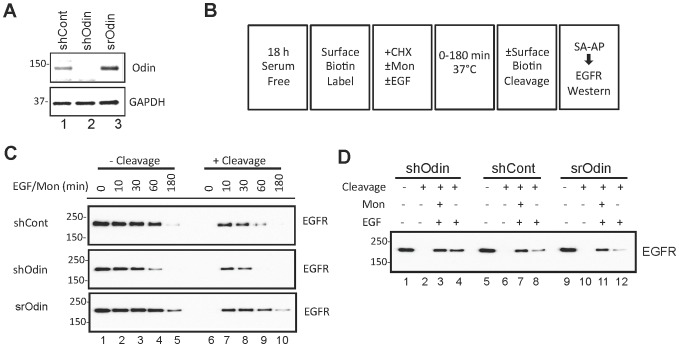
Effect of Odin on EGFR recycling. A, Western blot analysis of the whole cell lysates from lentivirus infected cells expressing a non-silencing control (shCont) or Odin-directed (shOdin) shRNA, or shOdin cells with stable ectopic expression of myc-Odin encoded by an shRNA-resistant Odin expression vector (srOdin). B, A schematic drawing showing from left to right the sequential experimental steps employed to measure EGFR degradation and internalization in shOdin, shCont and srOdin cells (monensin, mon; CHX, cyclohexamide, streptavidin affinity purification, SA-AP). C, Time-course of EGF stimulation of cells with basal (shCont), knocked down (shOdin) or rescued/over-expressed levels of Odin expression. Biotinylated EGFR was captured by using streptavidin (SA) beads, and then western blotted for EGFR. When cell surface biotin was removed prior to the SA adsorption step (+ cleavage), only internalized (i.e. protected from cleavage) EGFR are expected to be recovered, whereas in the absence of cleavage (- cleavage) both internalized and plasma membrane-localized EGFR would be captured. The cells were treated with monensin (Mon) to block receptor recycling. D, The effect of monensin on the amount of intracellular EGFR. Western blot analysis of biotin-labeled EGFR (captured by immobilized streptavidin) with indicated cleavage, EGF, and monensin treatment for 10 min. Results shown are representative of three independent experiments.

### EGFR Recycling as a function of Odin expression

To ensure the effects of Odin knockdown were not a consequence of off-target effects, a stable line derived from shOdin was established in which Odin protein expression was “rescued” by using an shRNA-resistant Odin expression vector (srOdin) (Fig. 6A). To further test whether Odin expression was affecting EGFR recycling, an assay was used to measure EGFR recycling as depicted schematically in Figure 6B [35].

In the absence of biotin cleavage (Fig. 6C, lanes 1–5), the streptavidin (SA)-captured biotinylated EGFR represent receptors originally on the cell surface at the zero time point, and including receptors that never internalized or those that internalized, but were not subjected to proteolytic degradation. Since the cells were simultaneously exposed to monensin, the assumption is made that receptor recycling was impaired. In shCont the EGFR signal began to decrease at 60 min, and with pronounced degradation by 180 min post-EGF. In the case of Odin knockdown (shOdin), EGFR were efficiently internalized and degradation was more rapid, with significant degradation by the 60 min time point. By contrast, when Odin expression was restored in the Odin knockdown cells to a level approximately 5-fold greater than normal endogenous levels (srOdin, See Fig. 6A), EGF receptors were stabilized relative to both the shOdin and shCont cells, with more EGFR signal remaining 180 min after EGF treatment than in the other two cell types (Fig. 6C, lanes 1–5).

Treatment of unstimulated cells with the cleavage solution abolished SA association with the EGFR demonstrating the complete removal of biotin moieties from surface-exposed EGFR (compared lanes 1 and 6, Fig. 6C). Therefore, following cleavage, the deduction is that biotinylated EGFR represent internalized receptors (Fig. 6C, lanes 7–10). A comparison of internalized biotinylated EGFR in the 3 cell types, representing basal (shCont), knockdown (shOdin) and recued/over-expressed levels of Odin (srOdin) indicated that the level of Odin expression is positively correlated with stabilization of internalized EGF receptors. The amount of internalized EGFR was similar at 10 min for each of the cell types, but diminished by 30 min and absent at 60 min post-EGF in shOdin cells (middle panel, lanes 7–10, Fig. 6C), whereas in srOdin, internalized EGFR were still detectable 180 min post-EGF (lower panel). The shCont cells, with an intermediate level of Odin compared to the other two cell types, showed a corresponding intermediate level of receptor stabilization, with some EGFR signal remaining at the 60 min time point (upper panel, lanes 7–10, Fig. 6C).

In a similar experiment, monensin treatment, which blocks receptor recycling to the plasma membrane [57], was conditional (Fig. 6D). In each of the 3 cell types, differing by Odin protein level, EGF stimulation for 10 min was associated with protection of the EGFR biotin adducts from cleavage as a consequence of efficient ligand-stimulated receptor internalization. At 10 min post-EGF, the total amount of internalized EGFR, calculated as the sum of internalized plus degraded EGFR, in shOdin, shCont, and srOdin was 68±4%, 56±6% and 57±8% (mean ±SD, n = 3), respectively, and not significantly different (i.e. p>0.05). In each of the cell types monensin treatment was associated with an accumulation of internalized EGFR relative to cells not exposed to monensin (compare lanes 3–4, 7–8, and 11–12 in Fig. 6D), and with the difference corresponding to the fraction of recycled receptors. The size of the fraction of recycled EGFR was proportional to the level of Odin expression, i.e. shOdin>shCont>srOdin, with a relatively small amount in cells with Odin knock down (shOdin, lanes 3 and 4, Fig. 6D), and a substantially greater population of recycled EGFR in the Odin over-expressing srOdin line (compare lanes 11 and 12, Fig. 6D), and in the shCont cells (lanes 7–8). To quantify EGFR recycling rates, the difference between the ±monensin values was determined relative to the total amount of internalized EGFR. For example, for EGFR in shOdin, as depicted in Fig. 6D: [(lane 3) – (lane 4)]/(lane 3). After 10 min EGF, the EGFR recycling rates were 56±7% for shOdin, which was statistically significantly less than that for shCont (80±5%) and srOdin (86±4%) (mean ±SD, n = 3, p<0.05).

### The effect of Odin on EGFR trafficking in a non-small cell lung carcinoma model

**Figure 7:**
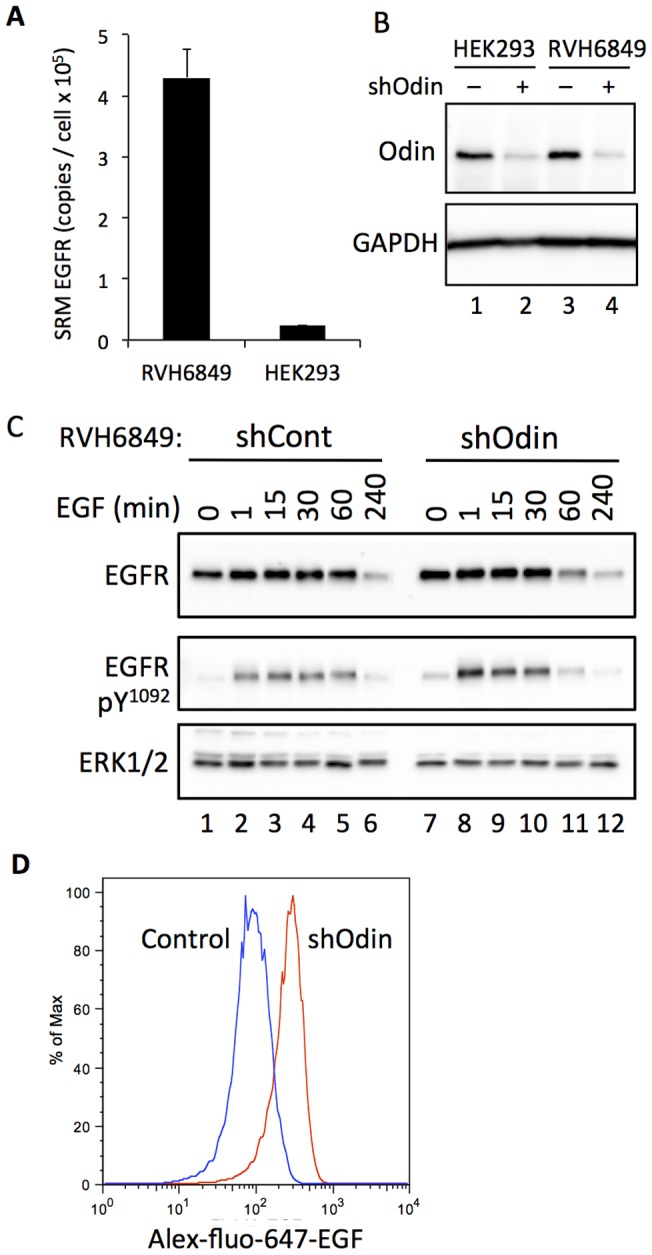
Effect of reduced Odin expression on EGFR cell surface localization and EGF-induced EGFR downregulation in non-small cell lung carcinoma (NSCLC) cells. A, The concentrations of endogenous wild type EGFR protein in HEK293 and RVH6849 were determined by using SRM-MS and a spiked-in heavy isotope labelled EGFR peptide (Table 1). B, Western blot analysis confirmed the efficient lentivirus/shRNA-mediated knock down of endogenous Odin protein expression in the two cell types, and compared with GAPDH as a loading control. C, RVH6849-shCont and RVH6849-shOdin were treated with EGF (10 ng/ml, 37°C) for the indicated durations, and then immuno-blotted with the indicated antibodies. The result shown is representative of three experiments. D, RVH6849 with (shOdin, red) or without (Control, blue) knock down of endogenous Odin were incubated with Alex-Fluo-647-conjugated EGF and then quantified by FACS according to the bound ligand as a measure of cell surface EGFR. SRM measurements (A) represent mean ±SE for 4 independent passages of the indicated cells.

We next examined the effect of knocking down Odin protein expression in the non-small cell lung carcinoma cell line RVH6849, which expresses wild type EGFR, but at a level more than 10-fold higher than HEK293 as measured by SRM-MS (Fig. 7A). Western blot analysis showed that there was similar endogenous Odin expression in HEK293 and RVH6849, and that Odin was effectively knocked down by shRNA in each of them (Fig. 7B). The rate of EGF ligand-stimulated EGFR downregulation was much slower in RVH6849 cells compared with HEK293 (Fig. 7C, lanes 1–6). EGF-stimulated EGFR downregulation was accelerated in RVH6849 cells when Odin expression was knocked down (Fig. 7C, lanes 7–12), with EGFR levels falling to approximately 50% by the 60 min time point, consistent with what was observed in the HEK293 background. By comparison, in the control cells expressing a non-silencing shRNA, EGFR downregulation was not appreciable 60 min after EGF, and was decreased to roughly 50% only after 4 h EGF treatment. In RVH6849 with or without Odin knockdown EGF-stimulated EGFR activation as measured by phosphorylation at Y^1092^ was rapidly achieved, in both cases reaching an approximate maximum by 1-min (Fig. 7C, middle panel). The level of tyrosine phosphorylation at Y^1092^ was slightly higher in the shOdin cells 1 min post-EGF (compare lanes 2 and 8, middle panel, Fig. 7C), but the signal, similar to that of the receptor itself, was more transient than in the control cells. For example, a comparison of the 60 min time points (lanes 5 and 11, middle panel, Fig. 7C) shows that the signal in shCont was similar to that seen at 30 min, whereas with shOdin the signal was considerably less. We were unable to stably transfect RVH6849 in order to assess the effects of Odin over-expression, and transiently transfected cells were largely rounded and non-adherent and therefore refractory to IF analysis. Therefore, in order to further gauge the effect of Odin on cellular EGFR in RVH6849, FACS was used to measure Alex-Fluo-647-EGF binding to intact RVH6849 cells with and without Odin knock down. This revealed an increased level of cell surface EGFR, as reflected in Alex-Fluo-647-EGF binding in cells lacking Odin (Fig. 7D).

## Discussion

We established that EGF-stimulated tyrosine phosphorylation of Odin occurs as an early event following ligand binding and independently of EGFR internalization or intracellular signaling to ERK (Fig. 1). This finding does not address whether Odin is a direct substrate of the activated receptor, but it suggests that Odin and an Odin tyrosine kinase(s) are proximal to the EGFR at the plasma membrane. EGFR activation and signaling to ERK kinases were not altered when Odin expression levels were modulated such that EGFR trafficking and stability were significantly altered, as discussed below. This is consistent with recent observations that intracellular signaling is initiated predominantly by plasma membrane-associated EGFR [58]. In contrast to the rapid tyrosine phosphorylation of Odin that preceded EGFR internalization, the slow accumulation of Odin tyrosine phosphorylation that followed EGFR activation is consistent with the kinetics of endocytosis-associated EGFR phosphorylations such as we reported previously for EGFR residues Y998 and S991 [19]. This study did not address requirements for Odin phosphorylation in EGFR trafficking, which is the focus of ongoing studies. However, our observations suggest the possibility that the trafficking fate of the EGFR may be governed by events such as Odin tyrosine phosphorylation that may occur prior to receptor internalization and downstream signal transduction. The tyrosine phosphorylation of Odin in response to the EGFR ligand TGFα, but not TNFα or anisomycin suggests that Odin tyrosine phosphorylation is a product of EGFR activation, rather than part of a cell stress response that may include EGFR internalization and recycling [35].

Despite its rapid initial phosphorylation downstream of EFG-stimulated receptor, a direct interaction between Odin and EGFR *in vivo* was not detected (data not shown), which is in agreement with other reports [36], [42]. Our SRM-MS analysis confirms the rather low intracellular concentration of Odin in HEK293 and RVH6849, which have EGFR expression levels that were measured to differ by more than 10-fold. This level of Odin expression is in general agreement with estimates from recent reports that comprehensively approximated intracellular protein abundances [52], [53]. By comparison, the recycling factor Rab11, which associates with and regulates recycling endosomes [59], was measured in these studies at levels approximately 100-fold higher than Odin [52], [53]. Our data indicate that Odin protein is present at levels one or more orders of magnitude below that of the EGFR (Fig. 2), suggesting that if Odin is indeed an adaptor protein, it may affect EGFR trafficking via low-stoichiometry and/or transient protein-protein interactions.

The levels of Odin protein expression dramatically affected the trafficking of activated EGF receptors. By comparison across the three intracellular Odin concentrations, spanning a range of at least 25-fold, we observed that internalized EGFR persisted for as little as 30 min in cells with reduced Odin levels, and as long as more than 3-h in cells with elevated Odin expression (Fig. 7). Both fluorescence microscopy imaging (Fig. 3) and biochemical measurements of EGFR downregulation (Fig. 5, 7), and of recycling (of surface-biotinylated EGFR; Fig. 6) support the conclusion that Odin is an effector of EGFR recycling. In HEK293 with elevated ectopic Odin expression, EGFR downregulation was delayed (Figures 3 and 5) and recycling increased (Figures 3 and 6). As summarized in Fig. 4, the Odin-mediated routing of internalized EGF receptors to a recycling endosome compartment was statistically significant and consistent with the biochemical measurements made by tracking internalized biotin-labeled EGFR (Fig. 6). Conversely, in both HEK293 and RVH6849, which represent a broad range of endogenous EGFR expression (Fig. 7A), Odin knock down produced more rapid EGFR downregulation. EGFR downregulation was generally slower in RVH6849 compared with HEK293 presumably as a consequence of their measured >10-fold higher EGFR protein level. In HEK293 lacking Odin there was less EGF-induced EGFR recycling, and consequently more trafficking of receptors to the lysosome compartment and degradation (Figures 3–6).Knockdown of Odin in RVH6849 caused an elevation in cell surface EGFR (Fig. 7D), suggesting that in the absence of Odin the pool of intracellular recycling EGFR was diminished, causing more of the receptors to be distributed to the cell surface. Indeed, surface receptors are known to constitutively internalize and recycle back to the cell surface [60]. By similar logic, we speculate the observed decrease in EGFR levels in HEK293 lacking Odin (Fig. 2D) may reflect EGFR downregulation due to serum and/or autocrine production of EGFR ligands, a phenomenon normally protected against by Odin-mediated recycling. The more pronounced apparent decrease in total EGFR levels associated with Odin knockdown in HEK293 compared with RVH6849 likely reflects the fact that the former express less than one-tenth the number of receptors than the later. Mann and colleagues noted a hyperproliferation phenotype in *odin^−/−^* MEFs [38]. Based on our findings, we speculate that in the absence of Odin-dependent receptor recycling, these cells may present an elevated density of cell surface-localized EGFR (and perhaps other Odin-regulated receptors) such that a stronger (than *wild type*) proliferative response is manifest upon exposure to ligand. Furthermore, we would predict that activated receptors would initiate signaling cascades even in the absence of Odin (as we observed), but would be prone to accelerated downregulation. This might explain why loss of Odin has not to our knowledge been observed as an oncogenic phenomenon.

In conclusion, our integrated proteomic, cell biology, and biochemical analysis of Odin and EGFR trafficking supports the conclusion that Odin is an effector of EGFR recycling. Our findings are consistent with the notion that Odin's ANK repeat region dictates membrane localization, and that its SAM and PTB domains effect interactions with trafficking factors and the EGFR. By governing the endocytic fate of internalized EGFR and possibly other receptors, Odin may enable cells to cope with receptor and ligand concentrations that are developmentally, physiologically, and pathologically highly variable. For example, Odin may prevent the elimination of receptors, and therefore preserve signaling potential in cells chronically exposed to low levels of ligands. Testing of these models will require further investigation.

## Supporting Information

Figure S1 **Quantification of Odin.** A GST-Odin fusion protein in known quantity was used as a standard to quantify Odin protein in samples by selected reaction monitoring mass spectrometry (SRM). A, MS/MS spectrum of the indicated Odin peptide. B, Stained gel showing purified GST and GST-Odin proteins. C, Measured SRM signals for the indicated GST and Odin peptides are shown.
(TIFF)

Figure S2 **The effect of Odin expression on transferrin receptor (TfR) level.** Whole cell lysates from HEK293 cells (Cont) or the same cell type stably expressing ectopic Odin (Odin), a control non-silencing shRNA (shCont), or an Odin-directed shRNA (shOdin) were prepared after treatment with transferrin (Tf, 5 µg/ml, 37°C) for the indicated durations and then subjected to western blot analysis with antibodies to TfR, or beta actin as a loading control. The result shown is representative of three experiments.
(TIFF)

Figure S3 **The effect of Odin expression on transferrin receptor (TfR) trafficking: The distribution of TfR in serum deprived Cont, Odin, shCont and shOdin cells.** The first column shows bright field or direct green fluorescence (which identifies lentivirus-infected cells). The cells were fixed and stained with TfR antibody (far red, second column) and DAPI to identify nuclei. Scale bar  = 6 µm.
(TIFF)

Figure S4 **The effect of Odin expression on transferrin receptor (TfR) trafficking: TfR endocytosis and co-localization with EEA1 after 30** **min transferrin (Tf) treatment.** The first column shows bright field or direct green fluorescence (which identifies lentivirus-infected cells). The cells were fixed and stained with TfR antibody (far red, second column). Cells were incubated with Tf (5 µg/ml) for 30 min and then fixed and stained with TfR antibody (far red, second column), and antibodies to EEA1 (red, third column). Pink indicate co-localization (Merge, fourth column); DAPI stain identifies nuclei. Scale bar  = 6 µm.
(TIFF)

Figure S5 **The effect of Odin expression on transferrin receptor (TfR) trafficking: TfR endocytosis and co-localization with Rab11 after 30** **min transferrin (Tf) treatment.** The first column shows bright field or direct green fluorescence (which identifies lentivirus-infected cells). The cells were fixed and stained with TfR antibody (far red, second column). Cells were incubated with Tf (5 µg/ml) for 30 min and then fixed and stained with TfR antibody (far red, second column), and antibodies to Rab11a/b (red, third column). Pink indicate co-localization (Merge, fourth column); DAPI stain identifies nuclei. Scale bar  = 6 µm.
(TIFF)

Figure S6 **The effect of Odin expression on transferrin receptor (TfR) trafficking.** The co-localization between TfR and EEA1 and Rab11a/b according to time of incubation with Tf (as shown in Figs. S3A, S3B, and S3C) was quantified by using Volocity software (Perkin Elmer). Mean co-localization coefficients (±SD, n = 6) represent pixel overlap between TfR and EEA1 or Rab11a/b.
(TIFF)
