# Supplementary figures and images for: Odin (ANKS1A) Modulates EGF Receptor Recycling and Stability
Source: PLoS One. 2013 Jun 25;8(6):e64817. doi: 10.1371/journal.pone.0064817 (PMC3692516; doi:10.1371/journal.pone.0064817)

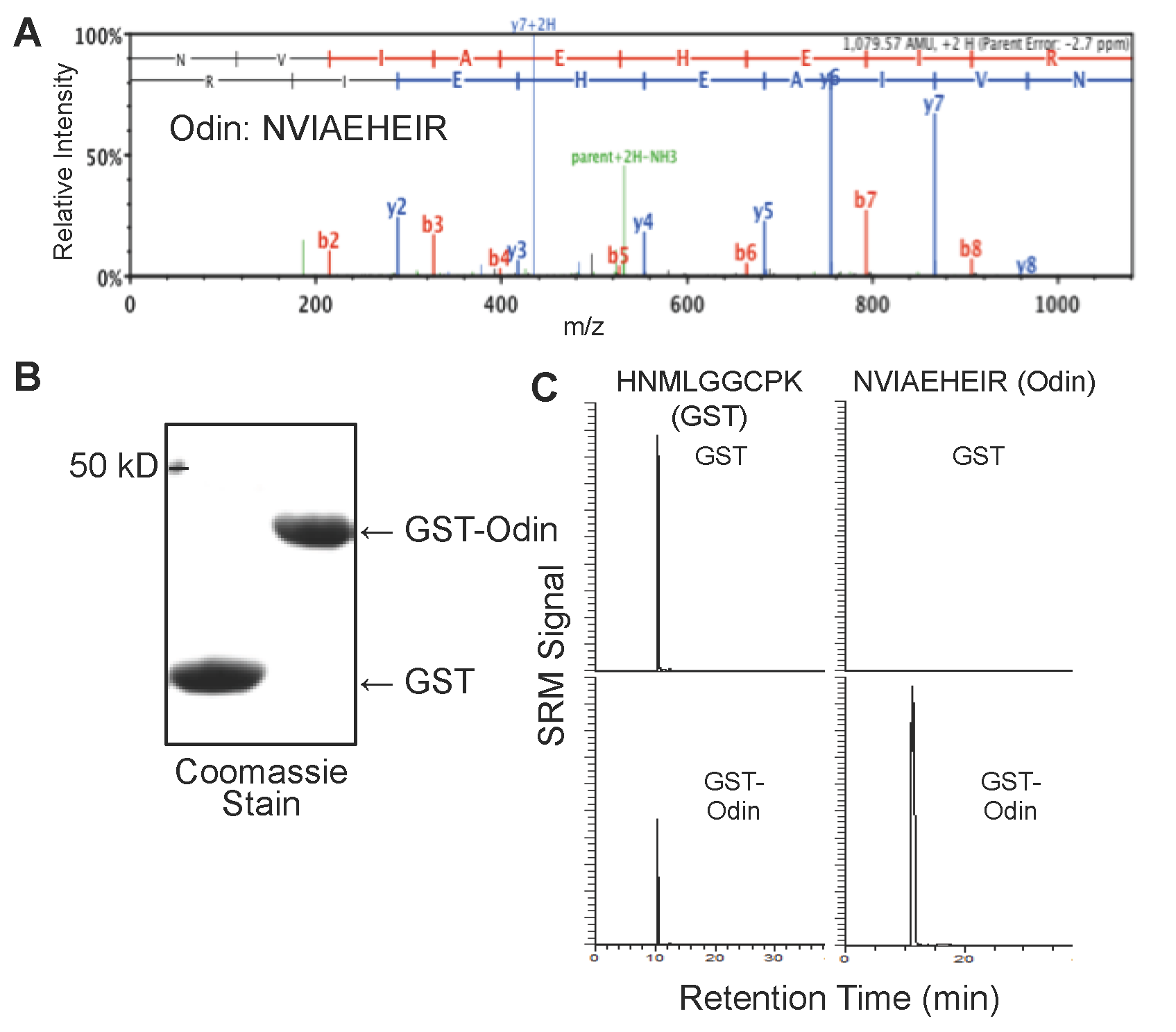

Supplement: Figure S1 — Quantification of Odin. A GST-Odin fusion protein in known quantity was used as a standard to quantify Odin protein in samples by selected reaction monitoring mass spectrometry (SRM). A, MS/MS spectrum of the indicated Odin peptide. B, Stained gel showing purified GST and GST-Odin proteins. C, Measured SRM signals for the indicated GST and Odin peptides are shown. (TIFF) [file pone.0064817.s001.tiff]

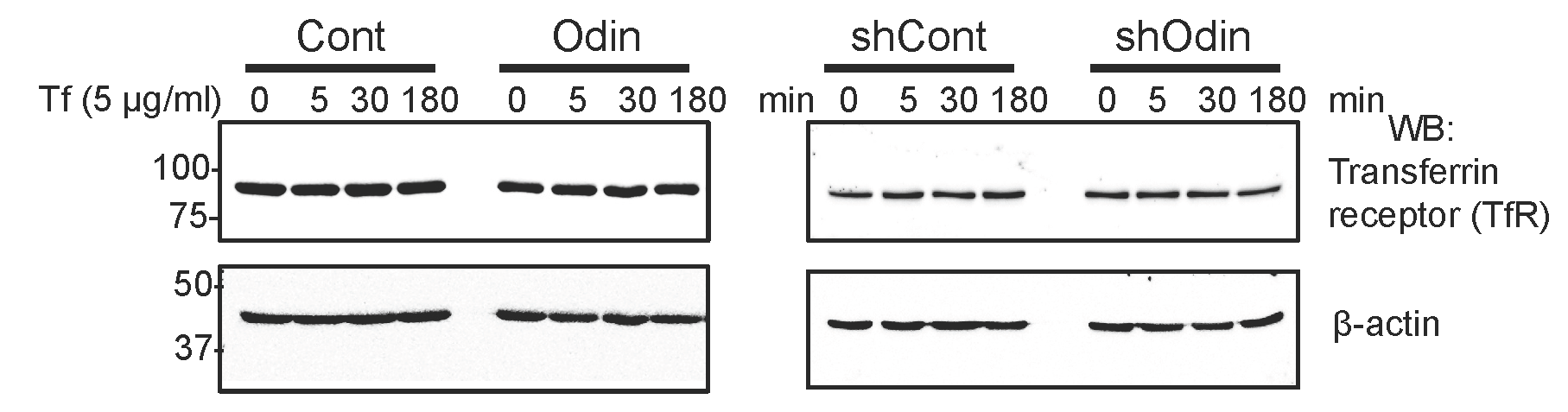

Supplement: Figure S2 — The effect of Odin expression on transferrin receptor (TfR) level. Whole cell lysates from HEK293 cells (Cont) or the same cell type stably expressing ectopic Odin (Odin), a control non-silencing shRNA (shCont), or an Odin-directed shRNA (shOdin) were prepared after treatment with transferrin (Tf, 5 µg/ml, 37°C) for the indicated durations and then subjected to western blot analysis with antibodies to TfR, or beta actin as a loading control. The result shown is representative of three experiments. (TIFF) [file pone.0064817.s002.tiff]

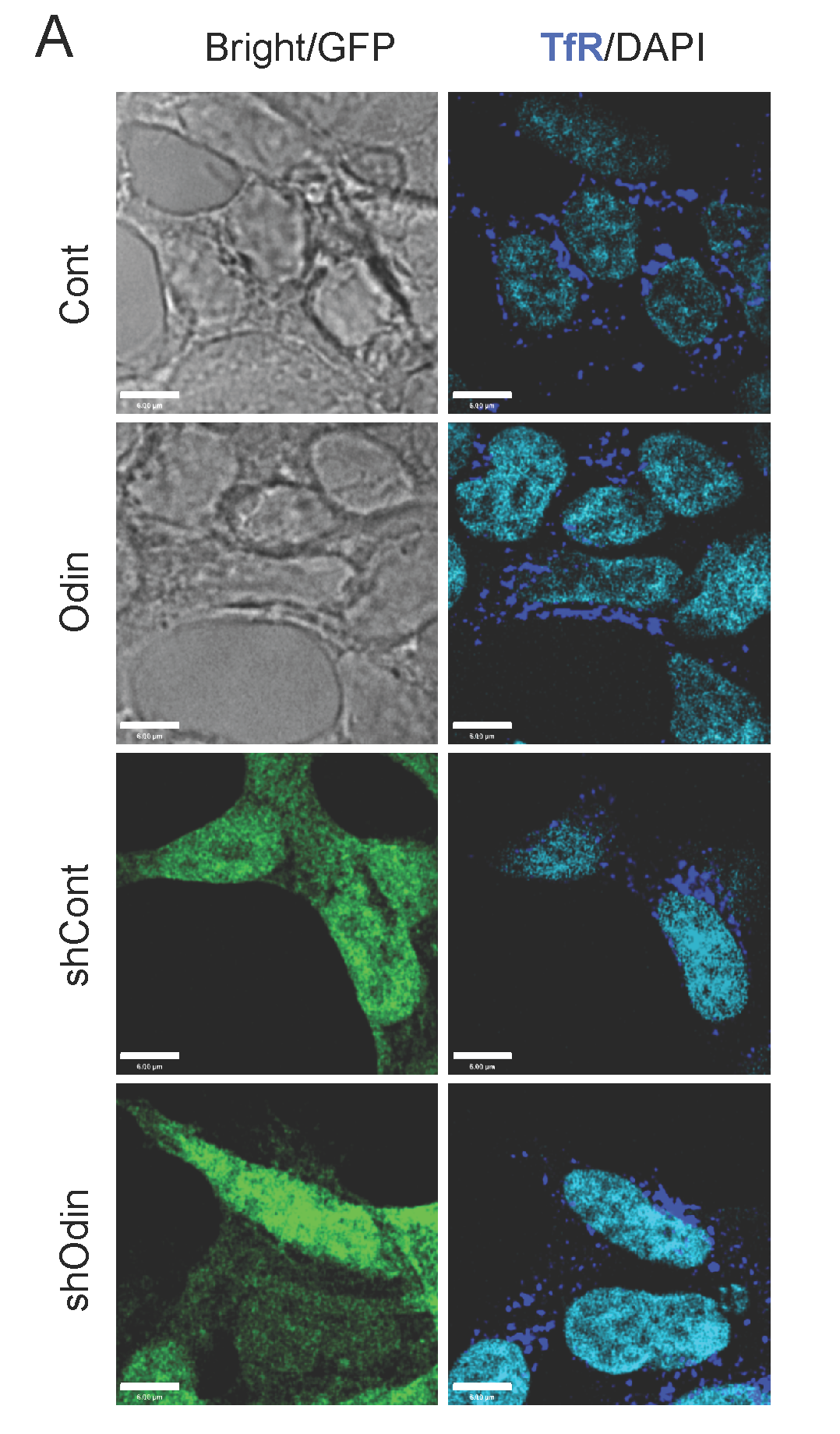

Supplement: Figure S3 — The effect of Odin expression on transferrin receptor (TfR) trafficking: The distribution of TfR in serum deprived Cont, Odin, shCont and shOdin cells. The first column shows bright field or direct green fluorescence (which identifies lentivirus-infected cells). The cells were fixed and stained with TfR antibody (far red, second column) and DAPI to identify nuclei. Scale bar = 6 µm. (TIFF) [file pone.0064817.s003.tiff]

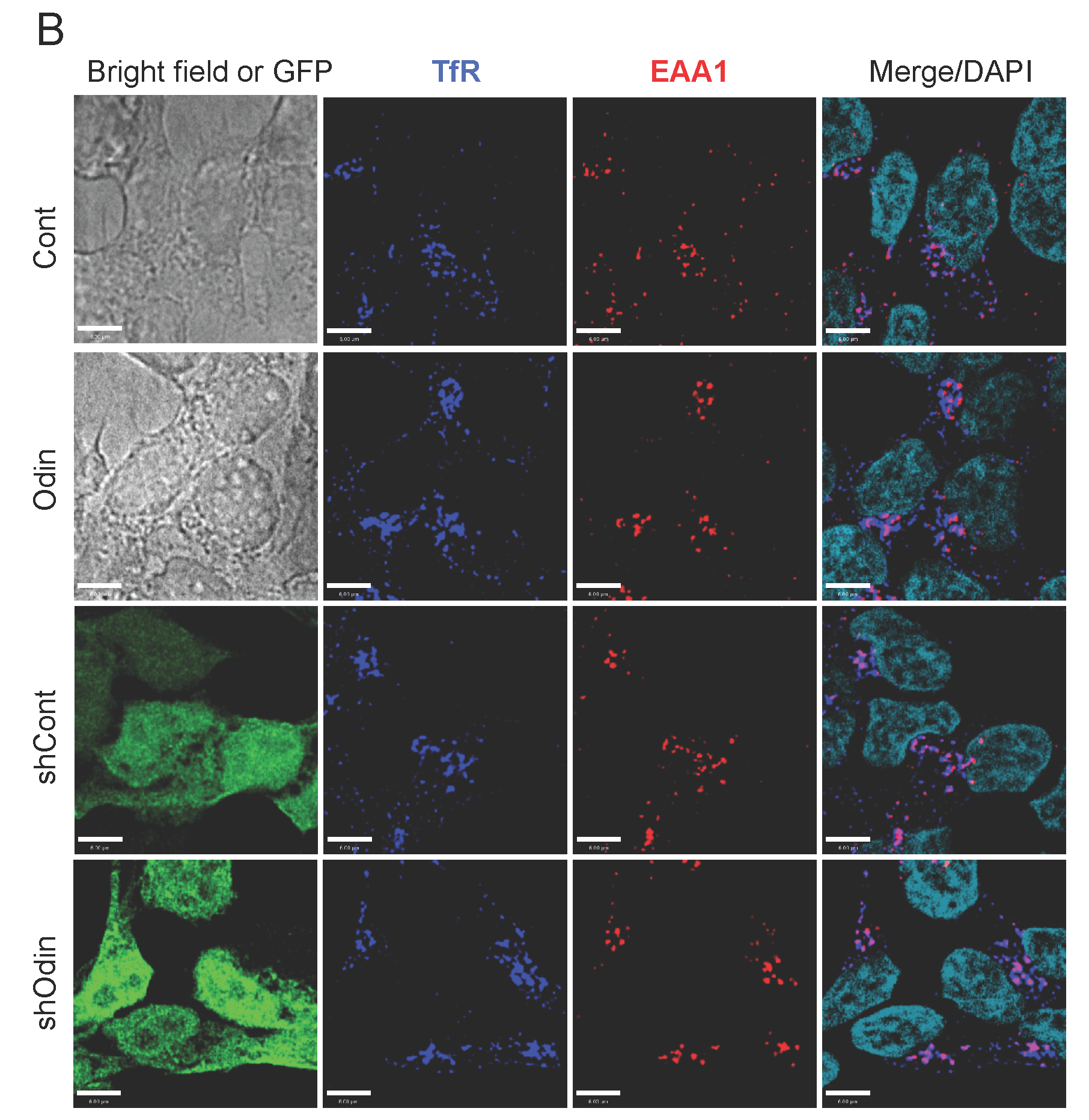

Supplement: Figure S4 — The effect of Odin expression on transferrin receptor (TfR) trafficking: TfR endocytosis and co-localization with EEA1 after 30 min transferrin (Tf) treatment. The first column shows bright field or direct green fluorescence (which identifies lentivirus-infected cells). The cells were fixed and stained with TfR antibody (far red, second column). Cells were incubated with Tf (5 µg/ml) for 30 min and then fixed and stained with TfR antibody (far red, second column), and antibodies to EEA1 (red, third column). Pink indicate co-localization (Merge, fourth column); DAPI stain identifies nuclei. Scale bar = 6 µm. (TIFF) [file pone.0064817.s004.tiff]

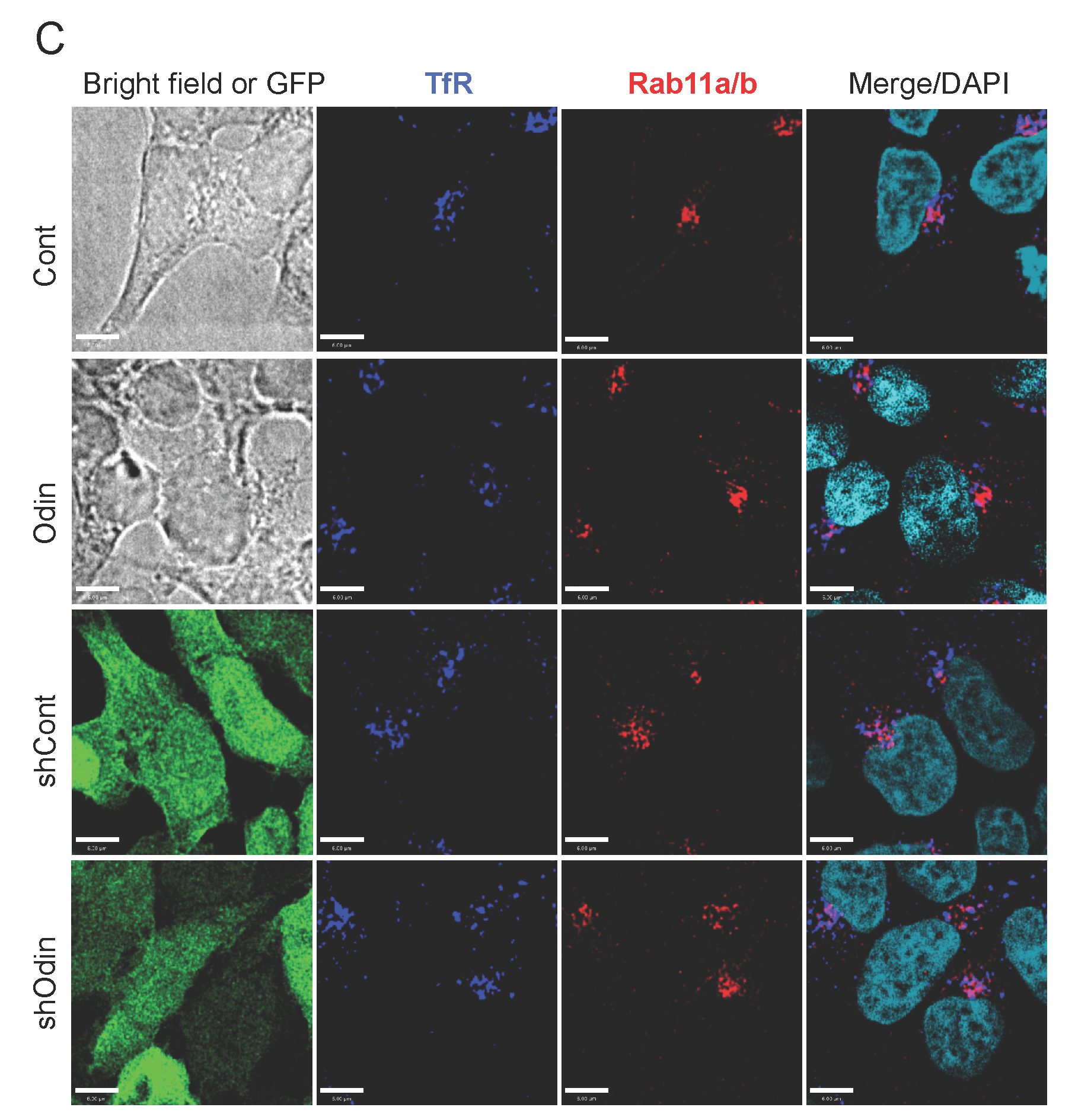

Supplement: Figure S5 — The effect of Odin expression on transferrin receptor (TfR) trafficking: TfR endocytosis and co-localization with Rab11 after 30 min transferrin (Tf) treatment. The first column shows bright field or direct green fluorescence (which identifies lentivirus-infected cells). The cells were fixed and stained with TfR antibody (far red, second column). Cells were incubated with Tf (5 µg/ml) for 30 min and then fixed and stained with TfR antibody (far red, second column), and antibodies to Rab11a/b (red, third column). Pink indicate co-localization (Merge, fourth column); DAPI stain identifies nuclei. Scale bar = 6 µm. (TIFF) [file pone.0064817.s005.tiff]

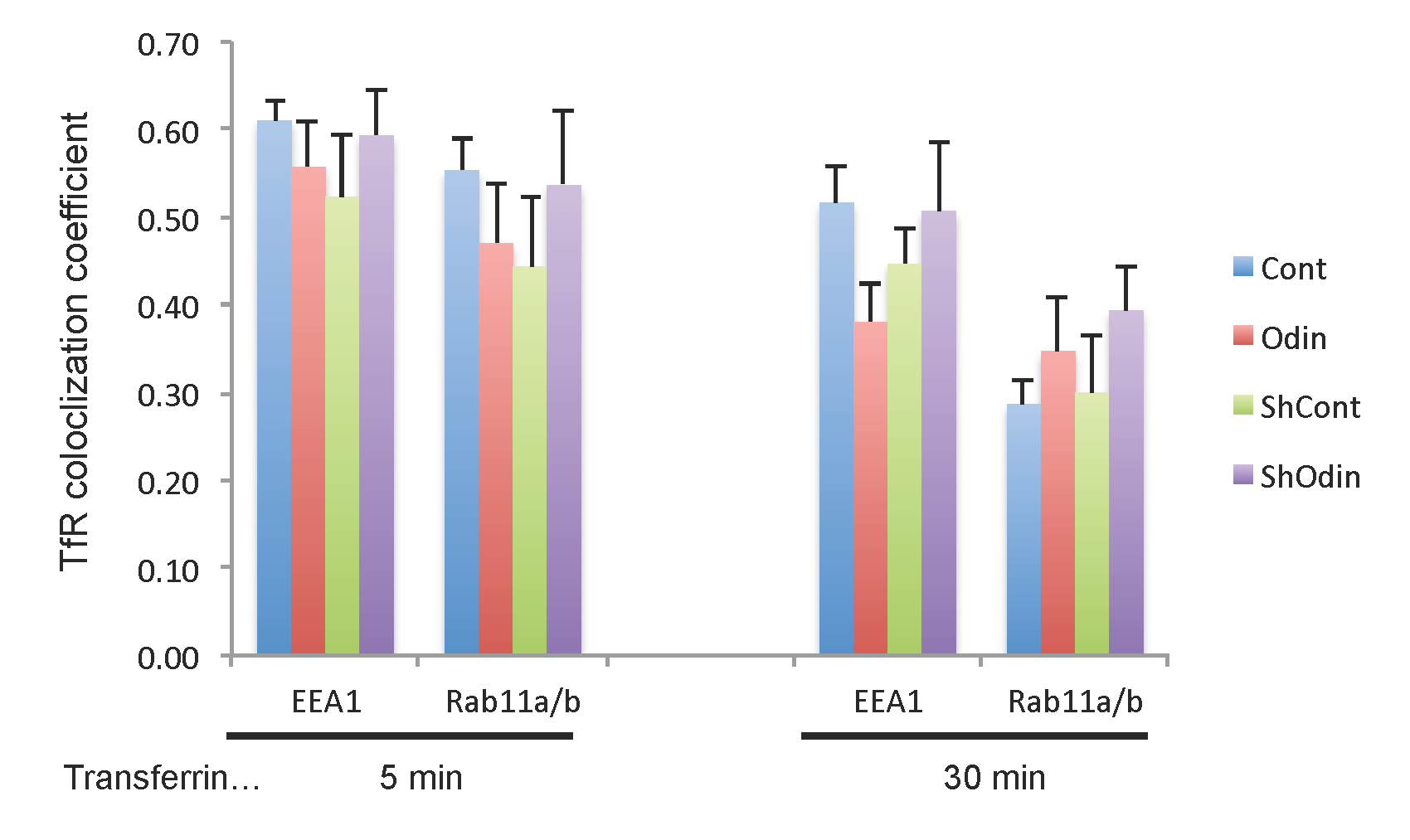

Supplement: Figure S6 — The effect of Odin expression on transferrin receptor (TfR) trafficking. The co-localization between TfR and EEA1 and Rab11a/b according to time of incubation with Tf (as shown in Figs. S3A, S3B, and S3C) was quantified by using Volocity software (Perkin Elmer). Mean co-localization coefficients (±SD, n = 6) represent pixel overlap between TfR and EEA1 or Rab11a/b. (TIFF) [file pone.0064817.s006.tiff]
